# Supplementary material for: Epidemiology and referral patterns of patients living with chronic kidney disease in Johannesburg, South Africa: A single centre experience
Source: PLOS Glob Public Health. 2024 Apr 18;4(4):e0003119. doi: 10.1371/journal.pgph.0003119 (PMC11034980; doi:10.1371/journal.pgph.0003119)
Supplement: Attachment — Submitted filename: Epidemiology and referral patterns of patients living with CKD in Johannesburg_Review.docx [file pgph.0003119.s001.docx]

Epidemiology and referral patterns of patients living with chronic kidney disease in Johannesburg, South Africa: a single centre experience

General comment

1. Well done to the authors for all the efforts to produce this manuscript and to publish
2. The referral patterns are needed to put in public health interventions for early referrals as there are measures to prevent progression.
3. It would be helpful to know how referral diagnosis differ from nephrologist diagnosis.
4. Effort should be made to report the proportions of late referrals and how they compare to other regions in Africa and globally.
5. It will help to report which cadre of health workforce do refer to the facility of the authors.
6. Some more details required for the study setting.

Title

7. Did the nephrologist agree to the referral diagnosis or are the diagnosis used the diagnosis of the attending nephrologist?

Abstract

8. Focus on late referrals and those who are referred with kidney failure should take centre stage

Introduction

9. I suggest authors do not start the write up with NCDs and I think it might be helpful to go straight to CKD and explore justification for the study based on what is known.
10. The burden on health imposed by CKD is especially prominent in low- and middle-income countries, [3],[5] with sub-Saharan Africa carrying a disproportionate burden of disease. is this high or low burden? Should come out clearer.
11. The prevalence of CKD in this region has been estimated to be 17.7%, [6]. I think it will be helpful to quote Stanifer et al 2014 also as a complete study than a subgroup analysis?

Methods

12. Some information from the hospital or study setting would be helpful – number of beds, number of patients per clinic, how many OPD days, number of nephrologists etc. which cadre of health workforce refer to the unit? Medical officers, nurses, specialist or even nephrologist? More information on study setting and referral patterns recommended
13. Why is this in inverted commas? “Chronic kidney disease” was broadly defined in accordance with the 2012 Kidney Disease: Improving Global Outcomes (KDIGO) guidelines.
14. The sample size was determined to be 960. How were these selected? what sampling method was used? This may introduce a lot of bias in the dataset.

Results

15. Not sure how the categorization of the other regions in table 1 would be useful. Can others keep it simple as nationals and non-nationals?
16. What proportion of patients required renal replacement on admission? Authors should seek to discuss this with regards to other studies in Africa and globally
17. Ascribed etiology in table 1 is this the nephrologists diagnosis or referral diagnosis? Would help to know how referral diagnosis differ from nephrologist diagnosis in the teaching hospital.
18. Some diagnosis in table 1 quite vague – what is nephrotoxins exposure or previous documented AKI? what is the real etiology?
19. “women formed a slight majority of patients in this group (51.3%) whist males were in the majority in non-Black ethnicities (56.4%, p = 0.024).” sentences like these should be well written to show comparison and read well.
20. A lot of sentences are repetition from the tables. Authors can save word count by referring to the table.
21. From table 3 About 30 patients were referred with stage 1 and 2 CKD. was wodering the indication for referral with normal renal function to the nephrologist. Nephrotic syndrome? what are the proportions of these then?
22. Was table 4 determined by logistic regression? Why is the 6^th^ column of Table 4 empty?

Discussion

23. the presence of elevated blood pressure may in these patients evidence underlying genetic or developmental pathophysiology such as *APOL1*-related kidney disease or reduced nephron endowment. This statement should be referenced
24. Whilst CKD prevalence is generally higher in women than in men, males are known to exhibit more rapid deterioration in renal function; this may account for the slight preponderance of males in this study. [29]. This statement can be justified if more males presented with CKD4 or 5 in your study. Is this the case. Then it helps to show.
25. Adequate control of hypertension appears more difficult to attain in males, [31]. is compliance not a factor. Fear of erectile dysfunction as side effects of anti hypertensives etc?
26. PLWH included in this study were more commonly female and of Black African ethnicity, mirroring the demographics of HIV infection in the general population. HIV incidence rates in South Africa are highest in the 15 – 29 year-old age group, Whilst prevalence rates are highest in women aged 30 – 49 years[32]. Any plausible reasons for this?
27. Late referral is a long-established barrier to improving dialysis-free survival the world over, with 30% of dialysis patients lacking significant pre-initiation nephrologist follow-up. [39]. It will help to compare late referral to others in the sub-region or Africa.
28. The advent of sodium-glucose 2 transporter (SGLT2) inhibitors offers the opportunity to substantially retard DKD progression and ameliorate the development of cardiovascular disease burden, with greater benefit being derived from earlier intervention facilitated by referral at lower CKD stages. [42]. Is this for only DKD? how well is SGLT2 used in SA? Are uptakes low and what may be the reasons for low uptakes?
