# Supplementary material for: Epidemiology and referral patterns of patients living with chronic kidney disease in Johannesburg, South Africa: A single centre experience
Source: PLOS Glob Public Health. 2024 Apr 18;4(4):e0003119. doi: 10.1371/journal.pgph.0003119 (PMC11034980; doi:10.1371/journal.pgph.0003119)
Supplement: Attachment — Submitted filename: Epidemiology_referral_CKD MCGB.docx [file pgph.0003119.s002.docx]

# **Epidemiology and referral patterns of patients living with chronic kidney disease in Johannesburg, South Africa: a single centre experience**

Yusuf Urade^1¶^, Zaheera Cassimjee ^1¶^, Chandni Dayal^1,2&^, Sheetal Chiba^1,2&^, Adekunle Ajayi^1,2&^, Malcolm Davies*^1,2¶^

^1^Department of Internal Medicine, University of the Witwatersrand, Johannesburg, Gauteng, South Africa

^2^Division of Nephrology, Helen Joseph Hospital, Johannesburg, Gauteng, South Africa

*Corresonding author

Email: [malcolm.davies@wits.ac.za](mailto:malcolm.davies@wits.ac.za)

^¶^YU, ZC, and MD developed the protocol for this study; YU undertook data collection; MD undertook data curation and analysis; YU, ZC, and MD wrote this paper

^&^CD, SC, and AA reviewed and edited this paper

Word count:

Abstract: 332

Main text: 2464

References:43

## **Abstract**

### **Introduction**

Chronic kidney disease (CKD) is a significant contributor to the global burden of non-communicable disease. Early intervention may facilitate slowing down of progression of CKD; recognition of at-risk patient groups may improve detection through screening. We report here on the epidemiology and referral patterns of CKD in the largest series of patients yet analysed in sub-Saharan Africa.

### **Methods**

A retrospective clinical records review of 960 patients attending a specialist nephrology outpatient clinic during the period 1 January 2011 – 31 December 2021 was undertaken. Demographic data, ascribed aetiology of kidney disease, comorbidities, and eGFR at referral were described for the cohort. Age and eGFR at referral were compared between ethnicities, sexes, and nationality categories using Mann Whitney U or Student t testing, as appropriate. Stepwise multivariate logistic regression was used to determine the effect of age, gender, ethnicity and immigration status on aetiological category of CKD and referral pattern.

**Results**

Black African patients comprised the most of the sample cohort and were younger at referral and more frequently female than other ethnicities; non-nationals were younger at referral than South Africans. Hypertension-associated kidney disease was the leading ascribed aetiological factor for CKD (40.7% of cases), followed by diabetic kidney disease (DKD) (19%), glomerular disease (12.5%), and HIV-associated kidney disease (11.8%). Hypertension-related (25.9%) and diabetic (10.7%) kidney diseases were not uncommon in people living with HIV. Advancing age and male sex increased the likelihood of diagnosis with hypertensive nephropathy, DKD and obstructive uropathy; males were additionally at increased risk of HIV-associated kidney disease and nephrotoxin exposure, as were patients of Black African ethnicity. Almost half (47.8%) of patients were referred with established CKD stage G4 or G5. Non-national immigration status and previous diagnosis with diabetes were associated with late referral; antecedent diagnosis with HIV reduced the odds of late referral.

#### **Conclusions**

Hypertension, diabetes, and HIV remain important aetiological factors in CKD. Despite the well-described risk of CKD in these disorders, referral to nephrology services occurs late. Interventions and policy decision actions targeting at-risk populations are required to improve referral practices.

Interventions and policy actions targeting at-risk populations are required to improve referral practices.

## **Keywords**

Chronic kidney disease, epidemiology, referral practices, HIV

**Abbreviations**

CKD: chronic kidney disease

CKD-EPI: Chronic Kidney Disease Epidemiology Collaboration

DKD: diabetic kidney disease

eGFR: estimated glomerular filtration rate

HIV: Human Immunodeficiency Virus

KF: kidney failure

KDIGO: Kidney Disease: Improving Global Outcomes

KRT: kidney replacement therapy

PLWH: people living with HIV

## **Main text**

### **Introduction**

Non-communicable diseases (NCDs) are major global contributors to premature mortality and morbidity and place significant strain on the allocation of healthcare resources. [1] Chronic kidney disease (CKD) is an important factor in adverse outcomes in patients living with NCDs due to the substantial increase in cardiovascular risk associated with this diagnosis. [2] The increasing global prevalence of CKD [3] mandates that healthcare programmes develop policy and infrastructure to address the growing patient population living with kidney dysfunction. [4]

The burden on health imposed by CKD is especially prominent in low- and middle-income countries, [3],[5] with sub-Saharan Africa carrying a disproportionate burden of disease. [3] The prevalence of CKD in this region has been estimated to be 17.7%, [6] exceeding the reported global average of 9.1%. [3] A complex interplay of genetic and socio-economic factors contribute to the increased risk of CKD development and progression in individuals of Black African ethnicity; [7],[8] accelerated onset of kidney failure (KF) has been reported in South African series. [9]

Despite the evident need, access to nephrology care in sub-Saharan Africa remains poor. [10],[11] South Africa boasts one of the few state-funded kidney replacement therapy (KRT) programmes in the region, [12] yet treatment access rates for patients living with KF reported for the country are amongst the lowest in the world. [13]

Early referral to specialist nephrology services slow down progression of CKD and reduces risk of premature cardiovascular death. [14] Public health initiatives and interventions at the sub-specialist level reduce incident CKD, [15] and may thus reduce strain on overburdened nephrologists in resource-limited settings such as sub-Saharan Africa.

Adequate capacitation of nephrology services and development of public health interventions require understanding of local epidemiology of CKD. Interrogation of referral patterns for patients living with CKD may additionally identify areas for improvement in sub-specialist care. Such data remains poorly described for sub-Saharan Africa. We therefore undertook the largest survey of patients diagnosed with CKD in South Africa to date with a view to better characterising CKD epidemiology and referral patterns.

### **Methods**

#### **Study population**

A retrospective file review of patients attending the Renal Outpatient Clinic at Helen Joseph Hospital (HJH) during the period 1 January 2011 – 31 December 2021 was undertaken. HJH is a tertiary-level facility which provides specialist nephrology services to the north-western suburbs of Johannesburg. The population served by the hospital is ethnically and socio-economically diverse. Black Africans make up 69.8% of residents compared to 22.9% for whites, 3.2% for Asians, and 2.9% for those of mixed ancestry; with 34.4% residing in informal housing. [16]

**Definitions**

“Chronic kidney disease” was broadly defined in accordance with the 2012 Kidney Disease: Improving Global Outcomes (KDIGO) guidelines; [17] that is, the presence of abnormality of kidney structure or function present for more than 3 months. To meet this definition, only those patients with documented follow-up of at least 3 months who demonstrated persistence of abnormality were considered for inclusion in this study.

CKD stage at referral was assigned as per eGFR as determined be the Chronic Kidney Disease Epidemiology Collaboration (CKD-EPI) equation and as defined by KDIGO. [17]

#### **Determination of sample size**

In-centre clinical activity reports were used to estimate the total number of patients on regular outpatient follow-up over the period of this study at 29040 individuals. Further analysis of clinic review rounds determined the average percentage of patients with documented CKD to be 80% per clinic. At a 95% confidence level and 2.5% margin of error, the representative sample size was thus determined to be 960 patients.

#### **Data extraction and storage**

Data pertaining to patient demographics (age at referral, sex, ethnicity, and country of origin); comorbid disease (hypertension, diabetes mellitus, and HIV status); ascribed aetiology of chronic kidney disease; and eGFR on referral were extracted anonymously from clinical records selected using convenience sampling and stored on a secured database prior to analysis using Stata® v. 17.0 (StataCorp, College Station, Texas). Clinical records were accessed and data extracted during the period 7/10/2022 – 26/12/2022.

#### **Statistical analysis**

Age and eGFR at referral were compared between ethnicities, sexes, and nationality categories using Mann Whitney U or Student t testing, as indicated. Stepwise multivariate logistic regression was used to determine the effect of age, gender, and ethnicity on aetiological category of CKD. Stepwise logistic regression modelling was used to evaluate the effect of demographics, immigration status, and comorbidity on referral patterns.

#### **Ethical considerations**

Permission to undertake this study was granted by the Human Research Ethics Committee (HREC) of the University of the Witwatersrand (certificate M220956); the study was undertaken in adherence with the principles of the Declaration of Helsinki. Formal consent to participate in this study was waived by the former in view of the retrospective nature of this study and in consideration of the preservation of data anonymity.

#### **Results**

Demographics, severity of renal dysfunction, comorbidities, and ascribed aetiology of CKD of the sample cohort are shown in Table 1.

| **Table 1. Demographics, referral characteristics, and ascribed aetiology of CKD in sample cohort (n = 960)** | |
| --- | --- |
| *Ethnicity**  Black African  Caucasian  Mixed ancestry  Asian | 641 (66.8%)  127 (13.2%)  117 (12.2%)  75 (7.8%) |
| *Sex**  Male  Female | 492 (51.3%)  468 (48.7%) |
| *Age at referral (years)*** | 57.2 ± 15.7 |
| *Nationality**  South African nationals  Non-nationals  African nations  Southern African region  Eastern African region  Western African region  Central African region  Southeast Asian nations | 850 (88.5%)  110 (11.5%)  105 (10.9%)  74 (7.7%)  19 (2.0%)  11 (1.1%)  1 (0.1%)  5 (0.5%) |
| *Severity of renal dysfunction on referral*  CKD-EPI eGFR (mL/min/1.73m^2^)^+^  CKD stage*  CKD stage G1  CKD stage G2  CKD stage G3a  CKD stage G3b  CKD stage G4  CKD stage G5 | 31 (17 – 48)  84 (8.8%)  50 (5.2%)  141 (14.7%)  226 (23.5%)  261 (27.2%)  198 (20.6%) |
| *Comorbidities*  Hypertension  HIV  Diabetes mellitus | 701 (73.0%)  309 (32.2%)  293 (30.5%) |
| *Ascribed aetiology of CKD*  Hypertension-associated kidney disease  Diabetic kidney disease  Glomerular diseases  HIV-associated kidney diseases  Obstructive uropathy  Nephrotoxin exposure  Autosomal dominant polycystic kidney diseasePrevious documented acute kidney injury  Congenital anomalies of genitourinary tract  Renovascular disease  Inherited tubulopathy  Cardiorenal syndrome | 391 (40.7%)  182 (19.0%)  120 (12.5%)  113 (11.8%)  64 (6.7%)  31 (3.2%)  31 (3.2%)  15 (1.6%)  8 (0.2%)  2 (0.2%)  2 (0.2%)  1 (0.1%) |
| **n (% of total N), **mean ± SD, ^+^median (interquartile range)* | |

### Variation in age at referral and sex were observed between ethnicities. Patients of Black African ethnicity were younger at referral than other ethnic groups (54.6 ± 15.4 years and 62.5 ± 15.1 years respectively, p < 0.001); women formed a slight majority of patients in this group (51.3%) whist males were in the majority in non-Black ethnicities (56.4%, p = 0.024). Non-nationals were younger at referral than South African nationals (48.9 ± 12.4 compared to 58.3 ± 15.8 years, p < 0.001); males were over-represented in the former group (60% compared to 50.1%, p = 0.051). Ascribed aetiology of CKD showed variation with demographic parameters. Increasing age was associated with higher likelihood of diagnosis with hypertension-associated kidney disease, diabetic kidney disease (DKD), and obstructive uropathy, but reduced the risk of diagnosis with glomerular disease; male sex was associated with increased risk of hypertension-associated kidney disease, DKD, HIV-associated kidney disease, obstructive uropathy, and nephrotoxin exposure. Black African ethnicity increased the risk of diagnosis with HIV-associated kidney diseases and CKD due to nephrotoxin exposure (Table 2).

| **Table 2. Effect of demographic parameters on ascribed aetiology of CKD** | | | |
| --- | --- | --- | --- |
|  |  | *Relative risk (95% CI)* | *p* |
| Age | Obstructive uropathy  Diabetic kidney disease  Hypertension-associated kidney disease  Glomerular disease | 1.05 (1.01 – 1.08)  1.04 (1.02 – 1.07)  1.03 (1.00 – 1.06)  0.94 (0.92 – 0.97) | 0.004  <0.001  0.022  0.002 |
| Male sex | Obstructive uropathy  Nephrotoxin exposure  Hypertension-associated kidney disease  Diabetic kidney disease  HIV-associated kidney diseases | 11.04 (4.04 – 30.22)  3.56 (1.14 – 9.85)  3.51 (1.52 – 8.08)  3.48 (1.47 – 8.25)  3.14 (1.29 – 7.67) | <0.001  0.027  0.003  0.005  0.012 |
| Black African ethnicity | HIV-associated kidney diseases  Nephrotoxin exposure | 5.91 (2.29 – 15.24)  3.35 (1.07 – 10.50) | <0.001  0.038 |

The 309 persons living with HIV (PLWH) included in the sample were more frequently of Black African than other ethnicity (88.0% compared to 12.0% for other ethnicities, p < 0.001); women contributed a larger proportion of this cohort than did males (55.7% versus 44.3%, p = 0.003).

| **Table 3. Demographics, referral characteristics, and ascribed aetiology of CKD in persons living with HIV (n = 309)** | |
| --- | --- |
| *Ethnicity**  Black African  Mixed ancestry  Caucasian  Asian | 272 (88.0%)  29 (9.4%)  6 (1.9%)  2 (0.6%) |
| *Sex**  Male  Female | 137 (44.3%)  172 (55.7%) |
| *Age at referral (years)*** | 54.0 ± 16.9 |
| *Nationality**  South African nationals  Non-nationals | 270 (87.4%)  39 (12.6%) |
| *Severity of renal dysfunction on referral*  CKD-EPI eGFR (mL/min/1.73m^2^)^+^  CKD stage*  CKD stage G1  CKD stage G2  CKD stage G3a  CKD stage G3b  CKD stage G4  CKD stage G5 | 37.7 ± 24.1  15 (4.9%)  15 (4.9%)  69 (22.3%)  89 (28.8%)  74 (23.9%)  47 (15.2%) |
| *Comorbidities*  Hypertension  Diabetes mellitus | 176 (57.0%)  40 (12.9%) |
| *Ascribed aetiology of CKD*  HIV-associated kidney diseases  Hypertension-associated kidney disease  Diabetic kidney disease in the setting of HIV  Glomerular diseases in the setting of HIV  Obstructive uropathy  Previous documented acute kidney injury  Autosomal polycystic kidney disease  Nephrotoxin exposure  Tenofovir nephropathy  Congenital anomalies of genitourinary tract  Inherited tubulopathy | 113 (36.6%)  80 (25.9%)  33 (10.7%)  24 (7.8%)  10 (3.2%)  8 (2.6%)  4 (1.3%)  3 (1.0%)  2 (0.6%)  2 (0.6%)  1 (0.3%) |
| **n (% of total N), **mean ± SD, ^+^median (interquartile range)* | |

A significant proportion of patients (47.8%) were referred with established CKD of stage G4 or G5. Increasing age, non-South African nationality, and diagnosis with comorbid diabetes mellitus increased the odds of referral with an eGFR below 30 mL/min/1.73m^2^; a trend towards later referral was also noted for males (p = 0.053). Conversely, PLWH were less likely to be referred with established CKD of lower eGFR (Table 4). Non-South African nationality remained a significant predictor of late referral when analysis was restricted to patients presenting in CKD stage G5 (OR 2.23, 95% CI 1.44 – 3.44, p < 0.001).

| **Table 4. Predictors of referral in CKD stages G4 or G5** | | |
| --- | --- | --- |
|  | *OR (95% CI)* | *p* |
| Age (years) | 1.01 (1.00 – 1.02) | <0.001 |
| Non-South African nationality | 2.05 (1.35 – 3.12) | <0.001 |
| Comorbid diabetes mellitus | 1.45 (1.08 – 1.96) | 0.015 |
| Comorbid HIV infection | 0.71 (0.53 – 0.95) | 0.019 |
| Black African ethnicity |  | 0.230 |
| Male sex | 1.29 (0.99 – 1.68) | 0.053 |

### **Discussion**

Although accumulating data evidences high prevalence rates of CKD in South Africa and the sub-Saharan region, [6],[18-20] little is known about disease characteristics and referral patterns in patients living with the condition. The present study confirms significant contribution from well-described risk factors including hypertension, diabetes mellitus, HIV infection, and advancing age to the development of CKD in the local context. Despite these known associations, patient referral at early, actionable stages of CKD appears to be poor, with older patients, diabetics, and non-nationals being particularly at risk of late referral.

Up to 48% of the South African population may be hypertensive; [21] the prevalence rate for type 2 diabetes mellitus has been estimated at 15%. [22] South Africa remains the global epicentre of the HIV pandemic with 13% of the population living with the virus. [16] Local studies have reported CKD prevalence rates of 21% in PLWH, [23] 18% in diabetics, [24] and 25% in hypertensive patients, [25] although considerable variation exists dependant on the definition of CKD used. The diagnosis of hypertension-associated kidney disease in a predominantly Black African population is, however, controversial. [26] Whilst hypertension is a known contributor to the progression of CKD, the presence of elevated blood pressure may in these patients evidence underlying genetic or developmental pathophysiologies such as *APOL1*-related kidney disease or reduced nephron endowment.

Rapid urbanization and significant structural socio-economic inequalities in the local context [27] conspire to create a milieu favourable to the progression of CKD. [28] Historical injustices render patients of Black African ethnicity most likely to experience these risk factors, which contributes to younger presentation with advanced CKD as observed in this study.

Whilst CKD prevalence is generally higher in women than in men, males are known to exhibit more rapid deterioration in renal function; this may account for the slight preponderance of males in this study. [29] Male sex is known to accelerate progression of DKD to KF, an association which may reflect the effect of androgenic hormones on inflammatory and fibrotic pathways. [30] Adequate control of hypertension appears more difficult to attain in males, [31] which may facilitate progression of hypertension-associated kidney disease and other forms of CKD. These factors may underlie the increased risk of DKD and hypertension-associated kidney disease in males observed in this study.

People living with HIV comprised nearly one-third of referrals to nephrology services in this cohort, almost three times the estimated prevalence rate of HIV in the broader South African population. [16] PLWH included in this study were more commonly female and of Black African ethnicity, mirroring the demographics of HIV infection in the general population. HIV incidence rates in South Africa are highest in the 15 – 29 year-old age group, whilst prevalence rates are highest in women aged 30 – 49 years. [32] PLWH in the present study tended to be older, reflective of improved survival in the era of universal access to antiretroviral therapy (ART). [33] Non-communicable diseases, including hypertension and diabetes, have been shown to be increasing in PLWH as that population ages. [34] Age-related changes in the disease profile of PLWH are appreciable in the present study where hypertension-associated kidney disease and DKD are important contributors to CKD in these patients.

Immigrants constitute an at-risk group for CKD; migrants from sub-Saharan Africa are especially at risk due to a combination of genetic, environmental, and healthcare system-related factors in their respective countries of origin. [12], [35] South African legal restrictions on the employment of non-nationals compel immigrants to seek unprotected employment in the informal sector, often as day labourers and at reduced wages; the country’s crumbling bureaucracy hinders formalization of residency status which might otherwise permit escape from these conditions. [36] The excessive physical labour and restrictions on standards of living which result are likely to accelerate CKD progression. This may explain the increasing risk of CKD with duration of emigration reported in other studies. [37] Difficulties in accessing the South African state healthcare system due to lack of immigration documentation and systemic xenophobia [38] may additionally have contributed to the tendency for referral with advanced CKD exhibited by non-nationals in the present study.

Overall, referral with advanced CKD was not uncommon in this cohort with nearly half of patients presenting with established CKD stage G4 or G5. Late referral is a long-established barrier to improving dialysis-free survival the world over, with 30% of dialysis patients lacking significant pre-initiation nephrologist follow-up. [39] Older age, non-Caucasian ethnicity, presence of comorbidities, and lack of medical insurance have been associated with increased probability of late referral. [39] Local data supports a lack of health system responsiveness at the primary care level for aging patients, especially in the state sector, which delays referral to specialist services such as nephrology. [40]

Increased risk of referral with advanced stage CKD in diabetics in this cohort is of particular concern. Local data suggests an increased probability of exclusion from state KRT programmes in diabetics due to cardiovascular comorbidity [41] which may have contributed to ennui in referral to nephrology. The advent of sodium-glucose 2 transporter (SGLT2) inhibitors offers the opportunity to substantially retard DKD progression and ameliorate the development of cardiovascular disease burden, with greater benefit being derived from earlier intervention facilitated by referral at lower CKD stages. [42] The experience of kidney disease-targeting interventions in HIV offers a potential roadmap to improve referral of diabetics in the local setting, as evidenced by reduced odds of referral at advanced CKD stage amongst PLWH in this study.

Taken together, these data shed light on current challenges in the management of kidney disease in South Africa. The tendency of the burden of CKD to fall upon younger Black South Africans dependant on the resource-limited state sector mandates that healthcare systems and policy should be developed to reduce late referral to nephrology services. Trends towards increased risk of exposure to nephrotoxins in this CKD-vulnerable population require public educational interventions. Referral systems for diabetics require urgent review to reduce late presentation and subsequent exclusion from KRT programmes. Whilst referral of PLWH does not appear to be subject to these limitations, improved survival of these patients in the era of universal access to ART is likely to increase demand on nephrology services as the prevalence of hypertension-associated kidney disease and DKD rise in this population. Evaluation of trends in the nephrologist workforce in South Africa show that the country lacks sufficient specialists to provide adequate care for the existing CKD population; expansion of nephrologist training programmes is required to meet the anticipated increase in demand. [43] Late presentation of non-nationals with advanced CKD is an additional cause for concern. Lack of nephrology services in their countries of origin and financial limitations on the ability of these immigrants to seek care elsewhere render these patients dependant on the South African state-funded healthcare system. [12] South Africa at present lacks clear guidelines on the provision of medical care to non-nationals living with CKD; [12] policy needs to be developed to assist this comparatively small and vulnerable population without further compromising access to care for South Africans.

There are limitations to this study. The single-centre nature of this work, and the urbanised cohort recruited, may limit generalizability to the broader South African and sub-Saharan context. Substantial reliance on clinical, rather than histological, diagnoses for non-glomerular disease may have resulted in over-representation of hypertension-associated kidney disease as an ascribed aetiological factor. The lack of albuminuria assay on referral limits full assessment of CKD stage as recommended by KDIGO guidelines. [17] However, since referrals to this unit characteristically rely on eGFR as the primary indication, this limitation does not materially compromise analysis of referral patterns.

### **Conclusions**

Hypertension, diabetes, and HIV remain important aetiological factors in CKD in South Africa. Despite the recognised association of these comorbidities with CKD, many patients are referred late in disease course with advanced kidney dysfunction. Rapid progression of CKD in patients of Black African ethnicity results in younger presentation with important ramifications for kidney health systems. Non-nationals constitute an at-risk population for the development of CKD; these patients are particularly vulnerable to delays in referral. Evidence from the referral of people living with HIV suggests that specialist interventions may improve early referral; improvements in referral of diabetic patients are especially required, and public education initiatives to reduce nephrotoxin exposure need to be considered. Finally, healthcare policy for non-nationals is urgently required to improve provision of nephrology care to these patients.

### **Acknowledgements**

The authors gratefully acknowledge the contribution of the Division of Nephrology at Helen Joseph Hospital nursing and administrative staff in accessing clinical records for data collection.

### **References**

1. Couser WG, Remuzzi G, Mendis S, Tonelli M. The contribution of chronic kidney disease to the global burden of major non-communicable diseases. Kidney Int 2011;80(2):1258-1270 doi 10.1038/ki.2011.368
2. Sarnak MJ, Levey AS, Schoolworth AC, Coresh J, Culleton B, Hamm LL, et al. Kidney disease as a risk factor for development of cardiovascular disease. Circulation 2008;108:2154-2169 doi 10.1161/01.CIR.0000095676.90936.80
3. GBD Chronic Kidney Disease Collaboration. Global, regional, and national burden of chronic kidney disease, 1990-2017: a systematic analysis for the Global Burden of Disease Study 2017. Lancet 2020;295(10225):709-733 doi 0.1016/S0140-6736(20)30045-3
4. Cockwell P, Fisher L-A. The global burden of chronic kidney disease. Lancet 2020;395(10225):662-664 doi 10.1016/S0140-6736(19)32977-0
5. Ke C, Liang J, Liu M, Liu S, Wang C. Burden of chronic kidney disease and its risk-attributable burden in 137 low- and middle-income countries, 1990-2019: results from the global burden of disease study 2019. BMC Nephrol 2022;23(17) doi 10.1186/s12882-021-02597-3
6. Kaze AD, Ilori T, Jaar BG, Echouffo-Tcheugui JB. Burden of chronic kidney disease on the African continent: a systematic review and meta-analysis. BMC Nephrol 2018;19(125) doi 10.1186/s12882-018-0930-5
7. Laster M, Shen JI, Norris KC. Kidney disease among African Americans: a population perspective. Am J Kidney Dis 2018;72(5 Suppl 1):S3-S7 doi 10.1053/j.ajkd.2018.06.021
8. Umeukeje EM, Young BA. Genetics and ESKD disparities in African Americans. Am J Kidney Dis 2019;74(6):811-821 doi h10.1053/j.ajkd.2019.06.006
9. Meremo A, Paget G, Duarte R, Bintabara D, Naicker S. Progression of chronic kidney disease among black patients attending a tertiary hospital in Johannesburg, South Africa. PLoS ONE 2023;18(2):e0276356 doi 10.1371/journal.pone.0276356
10. Barsoum RS, Khalil SS, Arogundade FA. Fifty years of dialysis in Africa: challenges and progress. Am J Kidney Dis 2015;65(3):502-512 doi 10.1053/j.ajkd.2014.11.014
11. Jones E, Rayner BL, Effa EE, Okpechi IG, Schmitz M, Heering PJ. Survey on available treatment for acute kidney injury in the South African Development Community: are we ready for zero deaths by 2025 in sub-Saharan Africa? BMJ Open 2019;9(8):e029001 doi 10.1136/bmjopen-2019-029001
12. Davies M, Cassimjee Z. Provision of long-term renal replacement therapy to non-nationals in South Africa. South Afr Med J 2021;111(7):615-619 doi 10.7196/SAMJ.2021.v111i7.15395
13. Thurlow JS, Joshi M, Yan G, Norris KC, Agodoa LY, Yuan CM, et al. Global epidemiology of end-stage kidney disease and disparities in kidney replacement therapy. Am J Nephrol 2021;52(2):98-107 doi 10.1159/000514550
14. Powe NR. Early referral in chronic kidney disease: an enormous opportunity for prevention. Am J Kidney Dis 2003;41(2):505-507 doi 10.1053/ajkd.2003.50111
15. Hallan SI, Ovrehus MA, Romundstad S, Rifkin D, Langhammer A, Stevens PE, et al. Long-term trends in the prevalence of chronic kidney disease and the influence of cardiovascular risk factors in Norway. Kidney Int 2016;90”665-673 doi 10.1016/j.kint.2016.04.012
16. Statistics South Africa. Census 2022. Available from https://www.statssa.gov.za/ [accessed online 17 May 2023]
17. Kidney Disease: Improving Global Outcomes (KDIGO) CKD Work Group. KDIGO 2012 clinical practice guideline for the evaluation and management of chronic kidney disease. Kidney Int Suppl 2013;3(4):1-150 doi 10.1038/kisup.2012.76
18. Matsha TE, Yako YY, Rensburg MA, Hassan MS, Kengne AP, Erasmus RT. Chronic kidney diseases in mixed ancestry South African populations: prevalence, determinants and concordance between kidney function estimators. BMC Nephrol. 2013;2(14):75 doi 10.1186/1471-2369-14-75
19. Mogueo A, Echouffo-Tcheugui JB, Matsha TE, Erasmus RT, Kengne AP. Validation of two prediction models of undiagnosed chronic kidney disease in mixed-ancestry South Africans. BMC Nephrol. 2015;4(16):94 doi 10.1186/s12882-015-0093-6
20. Adeniyi AB, Laurence CE, Volmink JA, Davids MR. Prevalence of chronic kidney disease and association with cardiovascular risk factors among teachers in Cape Town, South Africa. Clin Kidney J 2017;10(3):363-369 doi 10.1093/ckj/sfw138
21. Kandala N-B, Nnanatu CC, Dukhi N, Sewpaul R, Davids A, Reddy SP. Mapping the burden of hypertension in South Africa: a comparative analysis of the national 2012 SANHES and the 2016 Demographic and Health Survey. Int J Environ Res Public Health 2021;18(10):5445 doi 10.3390/ijerph18105445
22. Pheiffer C, Pillay-van Wyk V, Turawa E, Levitt N, Kenge AP, Bradshaw D. Prevalence of type 2 diabetes in South Africa: a systematic review and metanalysis. Int J Environ Res Public Health 2021;18(11):5868 doi 10.3390/ijerph18115868
23. Kamkuemah K, Kaplan R, Bekker L-G, Little F, Myer C. Renal impairment in HIV-infected patients initiating tenofovir-containing antiretroviral therapy regimens in a primary healthcare setting in South Africa. Trop Med Int Health 2015;20(4):518-526 doi 10.1111/tmi.12446
24. Pillay S, Aldous C, Mahomed F. A deadly combination – HIV and diabetes mellitus: where are we now? South Afr Med J 2016;106(4):378-383 doi 10.7196/samj.2016.v106i4.9950
25. Rayner B, Becker P. The prevalence of microalbuminuria and ECG left ventricular hypertrophy in hypertensive patients in private practices in South Africa. Cardiovasc J S Afr 2006;17(5):2450249 PMD: 17117229
26. Freedman BI, Sedor JR. Hypertension-associated kidney disease perhaps no more. J Am Soc Nephrol 2008;19(11):2047-2051
27. Statistics South Africa. Mid-year population estimates 2021. Available from <http://www.statssa.gov.za/publications/P0302/> [accessed online 29/5/2023]
28. Jagannathan R, Patzer RE. Urbanization and kidney function decline in low and middle income countries. BMC Nephrol 2017;18(1):276 doi 10.1186/s12882-017-0685-4
29. Brar A, Markell M. Impact of gender and gender disparities in patients with kidney disease. Curr Opinion Nephrol Hypertens 2019;28(2):178-182 doi 10.1097/MNH.0000000000000482
30. Conte C, Antonelli G, Melica ME, Tarocchi M, Roagnani P, Peired AJ. Role of sex hormones in prevalent kidney diseases. Int J Mol Sci 2023;24(9):8244 doi 10.3390/ijms24098244
31. Minutolo R, Gabbai FB, Agarwal R, Garofalo C, Borrelli S, Chiodini P, et al. Sex difference in ambulatory blood pressure control associates with risk of ESKD and death in CKD patients receiving stable nephrology care. Nephrol Dial Transplant 2021;26(11):2000-2007 doi 10.1093/ndt/gfab017
32. Vandormael A, Akullian A, Siedner M, de Oliveira T, Barnighausen T, Tanser F. Declines in HIV incidence in a South African population-based cohort. Nat Commun 2019;10:5483 doi 10.1038/s41467-019-13473-y
33. Gona PN, Gona CM, Ballout S, Rao SR, Kimokoti K, Mapoma CC, Mokdad AH. Burden and changes in HIV/AIDS morbidity and mortality in Southern Africa Development Community countries, 1990-2017. BMC Public Health 2020;20:867 doi 10.1186/s12889-020-08988-9
34. Magodoro IM, Okello S, Dungeni M, Castle AC, Mureyani S, Danaei G. Association between HIV and prevalent hypertension and diabetes mellitus in South Africa: analysis of a nationally representative cross-sectional survey. Int J Infect Dis 2022;121:217-225 10.1016/j.ijid.2022.05.035
35. Crews DC. Greater burden of ESRD among immigrants: kwa nini? J Am Soc Nephrol 2018;29(7):1789-1790 doi 10.1681/ASN.2018050503
36. Theodore N, Pretorius A, Blaauw D, Schenck C. Informality and the context of reception in South Africa’s new immigrant destinations. Pop Space Place 2019;24(3):e2119 doi 10.1002/psp.2119
37. Dawson AZ, Garacci E, Ozieh M, Walker RJ, Egede LE. The relationship between immigration status and chronic kidney disease risk factors in immigrants and US-born adults. J Immigrant Minority Health 2020;22:1200-1207 doi 10.1007/s10903-020-01054-x
38. Abaerei A, Ncayiyana J, Levin J. Health-care utilization and associated factors in Gauteng province, South Africa. Global Health Action 2017;10:1 doi 10.1080/16549716.2017.1305765
39. Baer G, Lameire N, van Biesen W. Late referral of patients with end-stage renal disease: an in-depth review and suggestions for further actions. Nephrol Dial Transplant Plus 2010;3(1):17-27 doi 10.1093/ndtplus/sfp050
40. Pletzer K, Phaswana-Mafuya N. Patient experiences and health system responsiveness among older adults in South Africa. Global Health Action 2012;4(1):18545 doi 10.3402/gha.v5i0.18545
41. Molaoa TT, Bisiwe FB, Ndlovu KCZ. End-stage kidney disease and rationing of kidney replacement therapy in the free state province, South Africa: a retrospective study. BMC Nephrol 2021;22:174 doi 10.1186/s12882-021-02387-x
42. Martinez-Castelao A, Soler MJ, Teruel JLG, Navarro-Gonzalez JF, Fernandez-Fernandez B, Moreno F de A, et al. Optimizing the timing of nephrology referral for patients with diabetic kidney disease. Clin Kidney J 2020;14(1):5-8 doi 10.1093/ckj/sfaa125
43. Kumashie DD, Tiwari R, Hassen M, Chikte UME, Davids MR. Trends in the nephrologist workforce in South Africa (2002 – 2017) and forecasting for 2030. PLoS ONE 2021;16(8):e0255903 doi 10.1371/journal.pone.0255903
