# Supplementary material for: Epidemiology and referral patterns of patients living with chronic kidney disease in Johannesburg, South Africa: A single centre experience
Source: PLOS Glob Public Health. 2024 Apr 18;4(4):e0003119. doi: 10.1371/journal.pgph.0003119 (PMC11034980; doi:10.1371/journal.pgph.0003119)
Supplement: Attachment — Submitted filename: Response to reviewers.docx [file pgph.0003119.s003.docx]

11 January 2024

The Editors and Reviewer Panel,

PLOS Global Public Health

Dear Mesdames and Sirs:

Re: Submission of revised original research paper entitled “Epidemiology and referral patterns of patients living with chronic kidney disease in Johannesburg, South Africa: a single centre experience”

We wish to thank the Reviewers for their thoughtful and considered suggestions revisions to our study which we feel have greatly improved the quality of the submitted work. We have amended the paper according to these suggestions as per the below (please note that page and line references refer to the document entitled “Revised manuscript with Track Changes”).

General comment

1. Well done to the authors for all the efforts to produce this manuscript and to publish
2. The referral patterns are needed to put in public health interventions for early referrals as there are measures to prevent progression.

- *We thank the Reviewers for their kind remarks. Data on access to nephrology care before the development of overt ESKD in South Africa and Africa as a whole is lacking. We hope that our work will add to those voices calling for interventions to address the growing number of patients in the developing world living with CKD.*

3. It would be helpful to know how referral diagnosis differ from nephrologist diagnosis.

- *Referrals were received from a broad range of healthcare workers with varying experience in kidney disease. Most referrals are made on the basis of reduced eGFR without an aetiology having been formally made. To improve accuracy of aetiological categories only those aetiologies as determined by the attending nephrologist were considered. This has been specified in the Methods (page 9, line 178 – 179) (see also comment 7. below)*

4. Effort should be made to report the proportions of late referrals and how they compare to other regions in Africa and globally.

- *“Late referral” lacks consistent definition in available literature. In the context of the present study, referral in CKD G4 or G5 may be considered as a “late referral”. The Discussion has been expanded with regards to available literature on late referral globally and in the African context (page 16 – 17 , lines 302 - 319)*

5. It will help to report which cadre of health workforce do refer to the facility of the authors.
   - *Referring units have been specified in the Methods subsection “Study site” (page 8 lines 153 – 156)*
6. Some more details required for the study setting.
   - *A detailed subsection on the study site has been added to the Methods (page 8 lines 144 - 156)*

Title

7. Did the nephrologist agree to the referral diagnosis or are the diagnosis used the diagnosis of the attending nephrologist?
   - *Ascribed aetiology of CKD diagnostic categories are as determined by the attending nephrologist. This has been specified in the Methods (page 9 line 178-179) (see also comment 3. above)*

Abstract

8. Focus on late referrals and those who are referred with kidney failure should take centre stage

- *Results section of the Abstract has been amended as suggested (page 3 line 43 - 45)*

Introduction

9. I suggest authors do not start the write up with NCDs and I think it might be helpful to go straight to CKD and explore justification for the study based on what is known.

- *Section on NCDs has been removed (page 6). Justification for this study is outlined in page 7, lines 121 - 131)*

10. The burden on health imposed by CKD is especially prominent in low- and middle-income countries, [3],[5] with sub-Saharan Africa carrying a disproportionate burden of disease. is this high or low burden? Should come out clearer.

- *Amended to clarify disproportionately high burden of disease (page 6 line 111)*

11. The prevalence of CKD in this region has been estimated to be 17.7%, [6]. I think it will be helpful to quote Stanifer et al 2014 also as a complete study than a subgroup analysis?

- *Stanifer et al has been referenced (page 6, line 112, reference 4)*

Methods

12. Some information from the hospital or study setting would be helpful – number of beds, number of patients per clinic, how many OPD days, number of nephrologists etc. which cadre of health workforce refer to the unit? Medical officers, nurses, specialist or even nephrologist? More information on study setting and referral patterns recommended

- *A detailed subsection on the study site has been added to the Methods (page 8, lines 144 – 156)*

13. Why is this in inverted commas? “Chronic kidney disease” was broadly defined in accordance with the 2012 Kidney Disease: Improving Global Outcomes (KDIGO) guidelines.

- *Inverted commas were used to indicate reference to accepted terminology. To prevent confusion these have been removed as suggested (page 8 line 159)*

14. The sample size was determined to be 960. How were these selected? what sampling method was used? This may introduce a lot of bias in the dataset.

- *Simple random sampling using the lottery method was used to select patient files for retrospective review. All included patients met the study definition for CKD, viz., persistent abnormality of kidney structure or function present for at least 3 months. “The subsection Data extraction and storage” has been expanded accordingly (page 9 lines 175 – 176) and resultant limitations are discussed in the appropriate section (Discussion, page 19, lines 360-361).*

Results

15. Not sure how the categorization of the other regions in table 1 would be useful. Can others keep it simple as nationals and non-nationals?

- *Table has been amended as suggested*

16. What proportion of patients required renal replacement on admission? Authors should seek to discuss this with regards to other studies in Africa and globally

- *The present study evaluates aetiologies and severity of CKD on referral to an outpatient clinic and does not therefore include inpatients admitted to this facility. Local resource constraints in state-funded dialysis units preclude dialysis initiation for CKD G5 without first evaluating transplant eligibility as an entrance criterion for KRT. Access to dialysis is further restricted for transplant eligible patients by limited dialysis “slots” so that even those patients meeting this criterion who require dialysis are unlikely to receive this treatment. The Discussion section has been expanded to reference late presentation and presentation with CKD G4 – G5 in Africa and globally, as well as local experience on KRT access for patients presenting with KF to the South African state sector (page 16 - 17, lines 302 - 319) (see also comment 4. above)*

17. Ascribed etiology in table 1 is this the nephrologists diagnosis or referral diagnosis? Would help to know how referral diagnosis differ from nephrologist diagnosis in the teaching hospital.

- *Ascribed aetiology of CKD is as per the attending nephrologist. This has been clarified in the Methods (“Data extraction and storage” subsection, page 9, line 178 - 179) and table 1 has been amended appropriately to indicate this. Since referrals are received from a broad range of healthcare workers with varying levels of experience of kidney disease (as per the expanded “Study site” subsection in the Methods, page 8, line 153 - 156), CKD aetiology diagnoses as per the referring practitioner were not analysed to ensure homogeneity of data (see also comments 3. and 7. above)*

18. Some diagnosis in table 1 quite vague – what is nephrotoxins exposure or previous documented AKI? what is the real etiology?

- *Table 1 has been expanded to include subcategories for these aetiological categories*

19. “women formed a slight majority of patients in this group (51.3%) whist males were in the majority in non-Black ethnicities (56.4%, p = 0.024).” sentences like these should be well written to show comparison and read well.

- *Sentence has been rewritten (page 12 line 203 - 206)*

20. A lot of sentences are repetition from the tables. Authors can save word count by referring to the table.

- *Tables have been re-referenced in the Results*

21. From table 3 About 30 patients were referred with stage 1 and 2 CKD. was wodering the indication for referral with normal renal function to the nephrologist. Nephrotic syndrome? what are the proportions of these then?

- *Overall, 84 patients were referred in CKD stage G1 (Table 1). The aetiology of renal dysfunction in the majority of these patients (65, 77.4% of CKD G1 patients) was indeed primary glomerular disease with smaller contributions from ADPCKD (6 patients), hypertensive nephropathy with relatively preserved eGFR (5 patients), obstructive uropathies (3 patients), congenital abnormalities of the urogenital tract (2 patients), HIV-associated glomerular kidney diseases (2 patients). One patient had documented renovascular disease with relatively persevered eGFR.*
- *Table 3 references patients living with HIV included in this study. The 30 patients in CKD 1 and 2 in this group do indeed predominantly comprise patients with glomerular diseases in the setting of HIV (13 patients with glomerular diseases in the setting of HIV, and 8 with HIV-associated kidney disease in the form of HIVAN for a total of 21 patients or 70% of this group). Local experience of patterns of glomerular disease in the setting of HIV has previously been documented elsewhere (Diana NE, Davies M, Mosiane P, Vermeulen A, Naicker S. Clinicopathological correlation of kidney disease in HIV infection pre- and post-ART rollout. PLoSONE 2022;17(5):e0269260).*

22. Was table 4 determined by logistic regression? Why is the 6^th^ column of Table 4 empty?

- *Table 4 has been corrected to specify logistic regression analysis and the missing OR has been added.*

Discussion

23. the presence of elevated blood pressure may in these patients evidence underlying genetic or developmental pathophysiology such as *APOL1*-related kidney disease or reduced nephron endowment. This statement should be referenced

- *Statement has been referenced (references 26 and 27)*

24. Whilst CKD prevalence is generally higher in women than in men, males are known to exhibit more rapid deterioration in renal function; this may account for the slight preponderance of males in this study. [29]. This statement can be justified if more males presented with CKD4 or 5 in your study. Is this the case. Then it helps to show.

- *A trend towards increased odds of presentation in CKD G4 or G5 was observed in multivariate analysis (table 4). The relevant section in the Discussion has been expanded to emphasize this finding. CKD G4 or G5 was indeed more frequent in males (55.5% of males presented with eGFR < 30 compared to 44.5% of females, p = 0.009 in Chi-square testing)*

25. Adequate control of hypertension appears more difficult to attain in males, [31]. is compliance not a factor. Fear of erectile dysfunction as side effects of anti hypertensives etc?

- *The discussion on BP control in males has been expanded and additional references included (page 15, lines 264 – 269, references 32 - 34). Briefly, reasons for sex discrepancy in BP control are debated and likely multifactorial. Lower rates of medication adherence in males (for a variety of reasons, including fear of side-effects) (reference 33) may play a role, but biological differences in hypertensive pathways between the sexes are also likely to be of importance (reference 32).*

26. PLWH included in this study were more commonly female and of Black African ethnicity, mirroring the demographics of HIV infection in the general population. HIV incidence rates in South Africa are highest in the 15 – 29 year-old age group, Whilst prevalence rates are highest in women aged 30 – 49 years[32]. Any plausible reasons for this?

- *Higher rates of HIV infection in young Black African women in South Africa reflect a complex interplay between biological, socio-behavioural, and epidemiological factors. Persistent inequalities in gender and racial power dynamics in South Africa arising from the country’s colonial and Apartheid past and the enforced migrant labour system introduced in those eras which has had a lasting impact on the nuclear family have been associated with increased risk-taking behaviour with age-disparate partners in young Black women. Since full discussion of these factors is above the scope of the present paper a brief explanation has been added (page 16, line 278 - 279) with appropriate referencing (reference 35)*

27. Late referral is a long-established barrier to improving dialysis-free survival the world over, with 30% of dialysis patients lacking significant pre-initiation nephrologist follow-up. [39]. It will help to compare late referral to others in the sub-region or Africa.

- *The Discussion section has been expanded to reference late presentation and presentation with CKD G4 – G5 in Africa and globally, as well as local experience on KRT access for patients presenting with KF to the South African state sector (page 16 - 17, lines 302 – 319) (see also comments 4. and 16. above)*

28. The advent of sodium-glucose 2 transporter (SGLT2) inhibitors offers the opportunity to substantially retard DKD progression and ameliorate the development of cardiovascular disease burden, with greater benefit being derived from earlier intervention facilitated by referral at lower CKD stages. [42]. Is this for only DKD? how well is SGLT2 used in SA? Are uptakes low and what may be the reasons for low uptakes?

- *SGTL2 inhibitors are not currently available in the state sector in South Africa and have not been been available in the private sector for very long. No data is yet available on prescribing patterns of SGLT2 inhibitors in the private sector. Use of SGLT2 inhibitors for non-diabetic kidney disease was only approved by the South African pharmaceuticals regulator towards the end of 2023.*

We thank the Journal and the Reviewers for the opportunity to resubmit our revised paper and look forward to receiving any other suggestions which may be forthcoming which will add to the quality of this study.

Yours faithfully,

The Authors.
